# Interleukin-6 expression contributes to lapatinib resistance through maintenance of stemness property in HER2-positive breast cancer cells

**DOI:** 10.18632/oncotarget.11471

**Published:** 2016-08-22

**Authors:** Wei-Chien Huang, Chao-Ming Hung, Ching-Ting Wei, Tsung-Ming Chen, Pei-Hsuan Chien, Hsiao-Lin Pan, Yueh-Ming Lin, Yun-Ju Chen

**Affiliations:** ^1^ The Ph.D. program for Cancer Biology and Drug Discovery, China Medical University and Academia Sinica, Taichung 404, Taiwan; ^2^ Graduate Institute of Cancer Biology, China Medical University, Taichung 404, Taiwan; ^3^ Center for Molecular Medicine, China Medical University and Hospital, Taichung 404, Taiwan; ^4^ Department of Biotechnology, Asia University, Taichung 413, Taiwan; ^5^ School of Medicine for International Students, I-Shou University, Kaohsiung 824, Taiwan; ^6^ Department of General Surgery, E-Da Hospital, Kaohsiung 824, Taiwan; ^7^ Department and Graduate Institute of Aquaculture, National Kaohsiung Marine University, Kaohsiung 811, Taiwan; ^8^ Department of Medical Research, E-Da Hospital, Kaohsiung 824, Taiwan; ^9^ Division of Colorectal Surgery, Department of Surgery, Kaohsiung Chang Gung Memorial Hospital and Chang Gung University College of Medicine, Kaohsiung 833, Taiwan; ^10^ Department of Biological Science & Technology, I-Shou University, Kaohsiung 824, Taiwan

**Keywords:** lapatinib, interleukin-6, HER2, resistance, breast cancer

## Abstract

Lapatinib is an inhibitor of human epidermal growth factor receptor 2 (HER2), which is overexpressed in 20-25% of breast cancers. Clinically, lapatinib has shown promising benefits for HER2-positive breast cancer patients; however, patients eventually acquire resistance, limiting its long-term use. In a previous study, we found that interleukin-6 (IL-6) production was increased in acquired lapatinib-resistant HER2-positive breast cancer cells. In the present study, we confirmed that lapatinib-resistant cells had elevated IL-6 expression and also maintained both stemness population and property. The increase in IL-6 was required for stemness property maintenance, which was mediated primarily through the activation of signal transducer and activator of transcription 3 (STAT3). Blocking IL-6 activity reduced spheroid formation, cell viability and subsequently overcame lapatinib resistance, whereas stimulation of IL-6 rendered parental cells more resistant to lapatinib-induced cytotoxicity. These results point to a novel mechanism underlying lapatinib resistance and provide a potential strategy to overcome resistance via IL-6 inhibition.

## INTRODUCTION

Breast cancer is the most common cancer in women worldwide and its incidence is increasing yearly. It can be classified into several subtypes according to the expression of several biomarkers. Overexpression of human epidermal growth factor receptor 2 (HER2, also known as Neu, ErbB2, EGFR2) is found in approximately 20-25% of breast cancer patients [1, 2]. HER2 is a member of HER/ErbB family and plays a critical role in the cancer progression via its receptor tyrosine kinase (RTK) activity [3]. HER2 is also a positive regulator of the cancer stem cell (CSC) population in HER2-positive breast cancers [4, 5]. HER2 overexpression increases, whereas HER2 inhibition decreases, the breast tumor initiating cell (BTIC) population *in vitro* [6]. Consistent with this finding, a positive correlation between HER2 expression and CSC frequency is also observed in human breast cancers [7]. These important features render HER2-positive breast cancer a highly malignant state with a poor prognosis [8, 9]. Blockage of HER2 activity with the kinase inhibitor lapatinib (GW572016, Tykerb^®^) has shown remarkable clinical efficacy in most patients with HER2-positive breast tumors [10, 11], but these patients eventually develop resistance. The mechanism underlying acquired resistance to lapatinib has not yet been completely elucidated [11–13].

The cytokine interleukin-6 (IL-6) was initially identified as a critical regulator of the immune response [14, 15]. However, it also activates downstream signaling pathways, such as the JAK/STAT pathway, to enhance tumor progression [16]. Elevated expression of IL-6 or its receptor are commonly found in many cancer types, including breast cancer, and are associated with poor prognosis [17, 18]. Furthermore, IL-6 expression renders tumor cells resistant to anti-cancer therapies [19–21]. Interestingly, Hartman et al., report that HER2 enhances *IL6* transcription, resulting in the activation of the IL-6/JAK/STAT3 autocrine loop, which plays a pivotal role in the carcinogenesis of HER2-positive breast cancer [22]. Up-regulation of IL-6 enhanced HER2-mediated mammosphere formation [23], as well as the tumorigenic conversion of mammary stem cells (CD44^hi^CD24^lo^), by activating Jagged-1/Notch-3 signaling [24, 25]. These findings indicate the critical role of IL-6 in BTIC expansion in HER2-positive breast cancer cells.

Our previous study showed that long-term treatment of lapatinib in breast cancer cells with or without HER2 expression enhances NF-κB activation and subsequently results in the expression of NF-κB downstream genes, including *IL6*. Furthermore, increased IL-6 production was observed in HER2-positive breast cancer cells with acquired lapatinib resistance [26]. However, it remains unclear whether increased IL-6 expression determines the sensitivity of HER2-positive breast cancer cells to lapatinib. In this study, we confirmed that acquired lapatinib-resistant HER2-positive breast cancer cells had elevated IL-6, and that elevated IL-6 maintained the stemness population and property within these resistant cells through the activation of signal transducer and activator of transcription 3 (STAT3). Blocking IL-6 activity reduced the BTIC population and cell viability of these resistant cells. However, stimulation of IL-6 made parental cells more resistant to lapatinib treatment. Taken together, these results provide evidence that, when the survival signal of breast cancer cells is switched from HER2 to IL-6 signaling, lapatinib resistance may be acquired. Moreover, blockage of IL-6 activity may be a potential strategy to overcome this resistance.

## RESULTS

### Maintenance of stemness population and property is observed within acquired lapatinib-resistant clones

**Figure 1:**
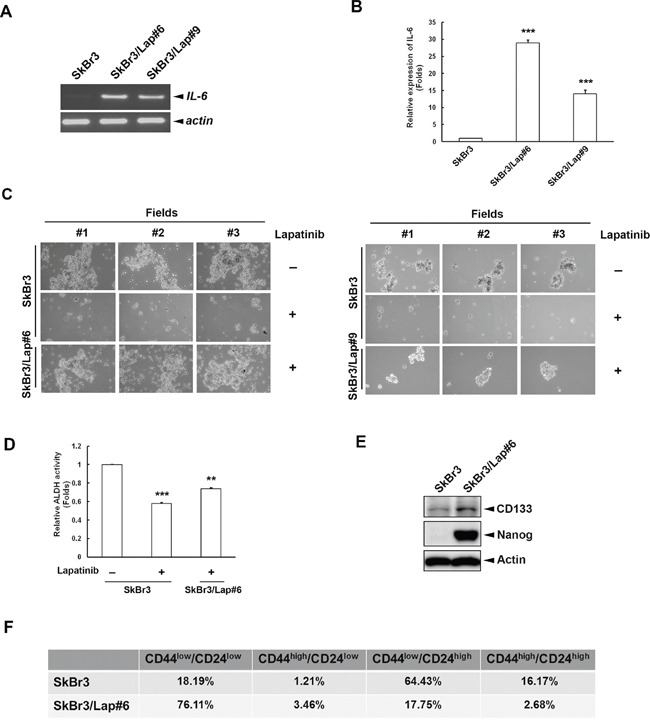
Maintenance of stemness population and property is observed within acquired lapatinib-resistant clones **A.** Total RNA was extracted from SkBr3, SkBr3/Lap#6 and SkBr3/Lap#9 cells and subjected to RT-PCR. mRNA expression of *IL6* and internal control *ACTIN* was examined by 1% electrophoresis. **B.** Media from SkBr3, SkBr3/Lap#6 and SkBr3/Lap#9 cells were collected to examine IL-6 production by ELISA. Statistical analysis was performed by Student's t test. ***, p<0.001 as compared to control group. **C.** SkBr3, SkBr3/Lap#6 and SkBr3/Lap#9 cells were cultured at a density of 2 × 10^4^ cells/ml in ultra-low attachment plates with spheroid-inducing medium. Cells were treated with 1 μM lapatinib for 7 days, and spheroid formation observed under microscope. **D.** SkBr3 and SkBr3/Lap#6 cells were treated with 1 μM lapatinib for 1 day, then re-seeded and 2 × 10^6^ cells per group were subjected to an ALDH activity assay. Statistical analysis was performed by Student's t test. **, p<0.01; ***, p<0.001 as compared to control group. **E.** The whole cell lysates of both SkBr3 and SkBr3/Lap#6 cells were harvested and subjected to western blot analysis with indicated antibodies. **F.** SkBr3 and SkBr3/Lap#6 cells were fixed and subjected to flow cytometry with indicated antibodies.

Our previous results indicated that long-term treatment of lapatinib enhances NF-κB-dependent gene expression, including the expression of *IL6* [26]. Here, we confirmed the elevation of *IL6* expression in HER2-positive breast cancer cells with acquired resistance to long-term lapatinib treatment. We found increased expression of *IL6* mRNA and IL-6 protein in two acquired lapatinib-resistant clones, SkBr3/Lap#6 and SkBr3/Lap#9 (Figures 1A and 1B). Since IL-6 plays a critical role in BTIC expansion in HER2-positive breast cancer cells, we next examined the stemness property of these resistant cells using spheroid formation and aldehyde dehydrogenase (ALDH) activity assays. As shown in Figure 1C, lapatinib treatment reduced spheroid formation in parental SkBr3 cells, whereas the ability to form spheroids was restored in both lapatinib-resistant SkBr3/Lap#6 (*left panel*) and SkBr3/Lap#9 (*right panel*) cells. Consistent with this observation, ALDH activity was also decreased in lapatinib-treated SkBr3 cells, but was maintained in SkBr3/Lap#6 cells (Figure 1D). The expression of stemness markers, CD133 and Nanog was also increased in SkBr3/Lap#6 cells (Figure 1E). Furthermore, the population of cancer stem cell marker CD44^high^/CD24^low^ expression was slightly elevated in SkBr3/Lap#6 cells as compared with that in SkBr3 cells (Figure 1F). These results suggest that IL-6 expression stemness property and population were maintained in acquired lapatinib-resistant cells.

### IL-6 is required for the maintenance of stemness property in acquired lapatinib-resistant cells

**Figure 2:**
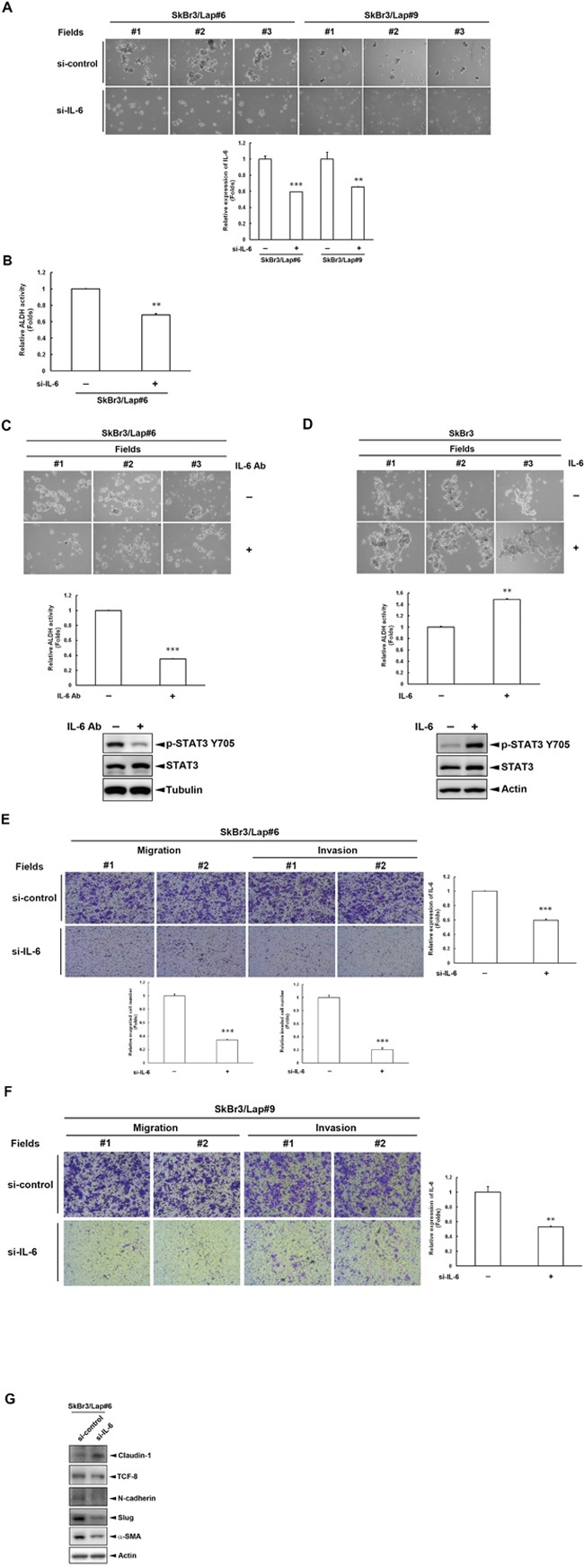
IL-6 is required for the maintenance of stemness property in cells with acquired lapatinib-resistance **A.** SkBr3/Lap#6 and SkBr3/Lap#9 cells were transfected with si-control or si-*IL6*. Two days later, cells were trypsinized and 4 × 10^4^ cells were cultured in ultra-low attachment plates with spheroid-inducing medium, followed by observation of spheroid formation under a microscope. Gene silencing of *IL6* was measured by ELISA. Statistical analysis was performed by Student's t test. **, p<0.01; ***, p<0.001 as compared to each control group. **B.** SkBr3/Lap#6 cells were transfected with si-control or si-*IL6*. Two days later, cells were trypsinized and 4 × 10^4^ cells were cultured in ultra-low attachment plates with spheroid-inducing medium for 7 days. Spheroids were collected and 2 × 10^6^ spheroids were subjected to an ALDH activity assay. Statistical analysis was performed by Student's t test. **, p<0.01 as compared to control group. **C.** SkBr3/Lap#6 cells at a density of 2 × 10^4^ cells/ml were cultured in ultra-low attachment plates with spheroid-inducing medium and treated with 10 μg/ml IL-6 antibody for 7 days, followed by observation of spheroid formation under a microscope. Then, 2 × 10^6^ spheroids were collected and subjected to an ALDH activity assay. Cell lysates from SkBr3/Lap#6 cells treated with 10 μg/ml IL-6 antibody were harvested and western blots used to examine phosphorylation of STAT3 using indicated antibodies. Statistical analysis was performed by Student's t test. ***, p<0.001 as compared to control group. **D.** SkBr3 cells at a density of 2 × 10^4^ cells/ml were cultured in ultra-low attachment plates with spheroid-inducing medium and treated with 20 ng/ml recombinant IL-6 protein for 7 days, followed by the observation of spheroid formation under microscope. Then, 2×10^6^ spheroids were collected and subjected to an ALDH activity assay. Cell lysates from SkBr3 cells treated with 20 ng/ml recombinant IL-6 protein were harvested and western blots used to examine phosphorylation of STAT3 using indicated antibodies. Statistical analysis was performed by Student's t test. **, p<0.01 as compared to control group. **E-F.** SkBr3/Lap#6 and SkBr3/Lap#9 cells were transfected with si-control or si-*IL6*. Two days later, cells were re-seeded and subjected to *in vitro* transwell migration or invasion assay for 48 hrs. Representative pictures of migrated or invaded cells were visualized by crystal violet staining and quantified. Gene silencing of *IL6* was confirmed by ELISA. Statistical analysis was performed by Student's t test. **, p<0.01; ***, p<0.001 as compared to control group. **G.** SkBr3/Lap#6 cells were transfected with si-control or si-*IL6*. Four days later, whole cell lysates were harvested and subjected to western blot analysis with indicated antibodies.

We next investigated whether IL-6 is required for the maintenance of stemness property in acquired lapatinib-resistant cells using an *IL6* siRNA and a neutralizing IL-6 antibody. As shown in Figure 2A, spheroid formation in SkBr3/Lap#6 and SkBr3/Lap#9 cells was attenuated (*upper panel*) when *IL6* expression was depleted by siRNA (*lower panel*). In addition, ALDH activity was also decreased in SkBr3/Lap#6 cells when *IL6* expression was blocked by siRNA (Figure 2B). Using an IL-6 neutralizing antibody to block IL-6 activity, there was attenuation in both spheroid formation (Figure 2C, *upper panel*) and ALDH activity (Figure 2C, *middle panel*) in SkBr3/Lap#6 cells. IL-6 activity was evaluated by examining its downstream signaling molecule, STAT3 tyrosine 705 phosphorylation (Figure 2C, *lower panel*). In contrast to these results, both spheroid formation (Figure 2D, *upper panel*) and ALDH activity (Figure 2D, *middle panel*) of parental SkBr3 cells were increased when cells were stimulated with recombinant IL-6 (Figure 2D, *lower panel*). Furthermore, the results from *in vitro* transwell assays showed that both migration and invasion of SkBr3/Lap#6 cells were blocked when *IL6* expression was silenced (Figure 2E). Similar results were also observed in SkBr3/Lap#9 cells (Figure 2F). The analysis of marker expression of epithelial-to-mesenchymal transition also revealed consistent results (Figure 2G). These results indicate that IL-6 activity is required for the maintenance of stemness property as well as migration/invasion in acquired lapatinib-resistant cells.

### STAT3 is the major downstream effector for IL-6-mediated maintenance of stemness property

**Figure 3:**
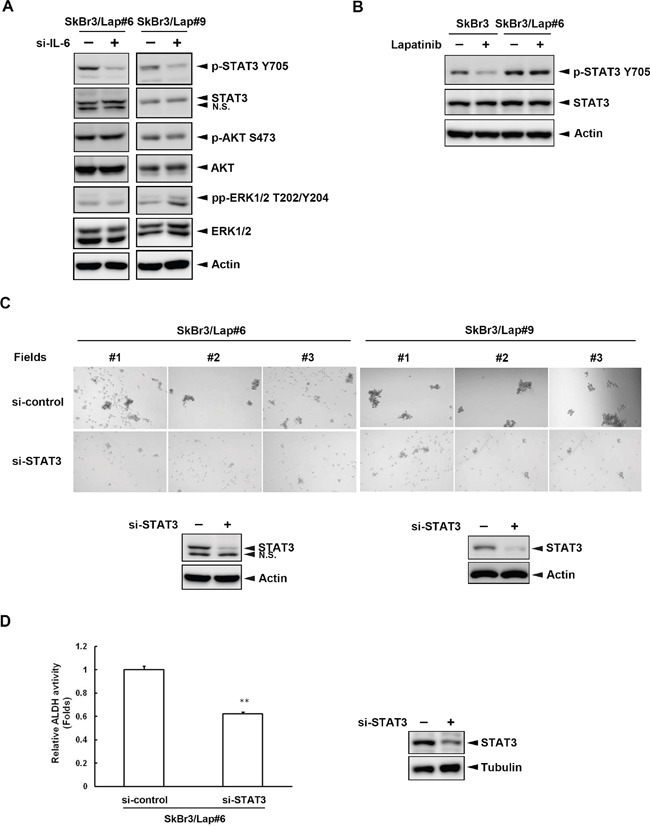
STAT3 activation is the major downstream effector for IL-6-mediated maintenance of stemness **A.** SkBr3/Lap#6 and SkBr3/Lap#9 cells were transfected with si-control or si-*IL6*. Four days later, cells lysates were harvested and the expressions of several IL-6 downstream effectors were examined by western blot with indicated antibodies. **B.** SkBr3 and SkBr3/Lap#6 cells were treated with 1 μM lapatinib for 24 hrs. Cell lysates were collected and the expressions of phosphorylated STAT3 tyrosine 705 and STAT3 were examined by western blot. **C.** SkBr3/Lap#6 and SkBr3/Lap#9 cells were transfected with si-control or si-STAT3. Two days later, cells were trypsinized and 4 × 10^4^ cells were cultured in ultra-low attachment plates with spheroid-inducing medium, followed by the observation of spheroid formation under microscope. Gene silencing of *STAT3* was examined by western blot. **D.** SkBr3/Lap#6 cells were transfected with si-control or si-*STAT3*. Two days later, cells were trypsinized and 4 × 10^4^ cells were cultured in ultra-low attachment plates with spheroid-inducing medium for 7 days. Then, 2 × 10^6^ spheroids were collected and subjected to an ALDH activity assay. Protein expression of STAT3 was examined by western blot. Statistical analysis was performed by Student's t test. **, p<0.01 as compared to control group.

IL-6 regulates biological responses by activating several intracellular signaling effectors, such as STAT3, ERK, and Akt. We further explored the molecular mechanism underlying IL-6-mediated effects in these lapatinib-resistant cells by examining the effects of silencing *IL6* on these downstream signaling effectors. Only tyrosine 705 phosphorylation of STAT3 was attenuated when SkBr3/Lap#6 cells lost *IL6* expression (Figure 3A, *left panel*). Similar results were also observed in SkBr3/Lap#9 cells (Figure 3A, *right panel*). Importantly, the tyrosine 705 phosphorylation of STAT3 in resistant cells was no longer altered by lapatinib treatment (Figure 3B). Next, we investigated whether STAT3 activation is required for IL-6-mediated regulation in these resistant cells. As shown in Figure 3C, when STAT3 expression was depleted by siRNA (*lower panel*), spheroid formation was decreased in both SkBr3/Lap#6 and SkBr3/Lap#9 cells (*upper panel*). Moreover, silencing of STAT3 expression reduced ALDH activity in SkBr3/Lap#6 cells (Figure 3D). These results suggest that STAT3 activation is the major downstream effector for IL-6-mediated maintenance of stemness in acquired lapatinib-resistant cells.

### IL-6 activity is critical for lapatinib resistance

**Figure 4:**
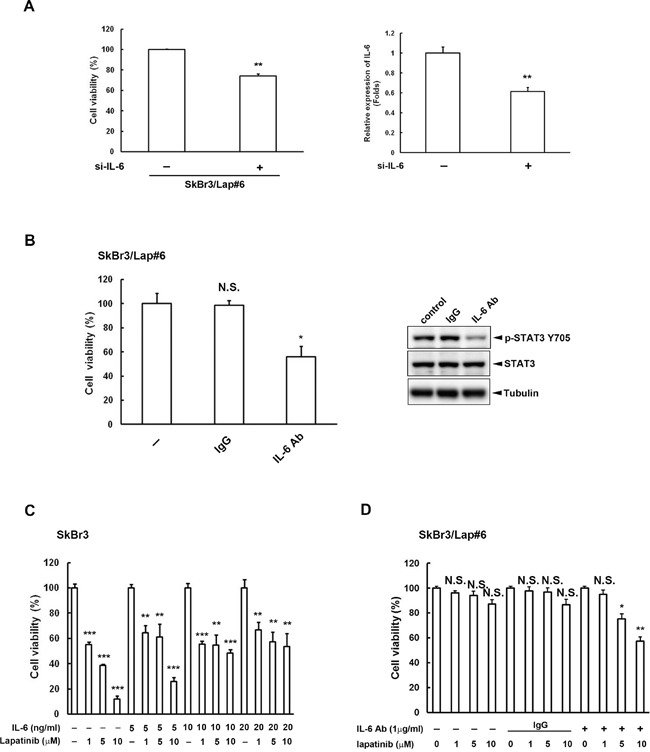
IL-6 activity is critical for lapatinib resistance **A.** SkBr3/Lap#6 cells were transfected with si-control or si-*IL6*. Two days later, cells were re-seeded at a density of 2 × 10^4^ in a 6-cm dish for 2 weeks. Cell viability was detected and quantified by crystal violet staining. Gene silencing of *IL6* was confirmed by ELISA. Statistical analysis was performed by Student's t test. **, p<0.01 as compared to control group. **B.** SkBr3/Lap#6 cells were cultured at a density of 1 × 10^4^ cells in 96-well plates with medium treated with either 10 μg/ml IgG or IL-6 antibody for 4 days. Cell viability was examined by MTT assay. Cell lysates treated with 10 μg/ml IL-6 antibody were also collected to examine the expression of phosphorylated STAT3 tyrosine 705 by western blot. Statistical analysis was performed by Student's t test. *, p<0.05 as compared to control group. N.S. represents non-significant. **C.** SkBr3 cells were pre-treated with different concentrations of recombinant IL-6 protein, followed by treatment with the indicated concentrations of lapatinib for 3 days. Cell viability was then examined by MTT assay. Statistical analysis was performed by Student's t test. **, p<0.01; ***, p<0.001 as compared to control group. **D.** SkBr3/Lap#6 cells were pre-treated with or without IL-6 antibody, followed by treatment with the indicated concentrations of lapatinib for 3 days. Cell viability was then examined by MTT assay. Statistical analysis was performed by Student's t test. *, p<0.05; **, p<0.01 as compared to each control group. N.S. represents non-significant.

To examine whether IL-6 is important for the survival of lapatinib-resistant cells, we inhibited IL-6 activity using both siRNA and neutralizing antibody. We found that the cell viability of lapatinib-resistant cells was decreased (Figure 4A, *left panel*) when *IL6* expression was blocked by siRNA (Figure 4A, *right panel*). Similar results were also observed when IL-6 activity was inhibited by antibody (Figure 4B). We also increased IL-6 activity in parental SkBr3 cells with the addition of recombinant IL-6 protein. The dose-dependent cytotoxicity by lapatinib in SkBr3 cells without recombinant IL-6 was gradually reversed when cells were treated with higher doses of recombinant IL-6 protein (Figure 4C). In contrast, SkBr3/Lap#6 cells became more sensitive to lapatinib treatment when IL-6 activity was inhibited by IL-6 antibody (Figure 4D). These results indicate that blocking IL-6 activity in resistant cells overcomes lapatinib resistance whereas stimulation of IL-6 activity in parental cells increases lapatinib resistance.

## DISCUSSION

Targeted therapies, such as HER2-targeted therapy in breast cancer, are designed to directly attack the oncogenic proteins expressed by cancer cells and have been successful anti-cancer therapies [27]. Two types of HER2-targeted therapy, lapatinib and trastuzumab (Herceptin®), are extensively used in clinic and show promising benefits for most HER2-positive breast cancer patients [10, 11]. However, patients eventually develop acquired resistance, even after initially responding well to these drugs. This is a serious problem challenging anti-HER2 therapy in the clinic, and the mechanisms underlying resistance to anti-HER2 therapy have been intensively investigated [11–13, 28]. In this study, we found that long-term treatment of lapatinib in HER2-positive breast cancer cells led to increased *IL6* expression, subsequent activation of downstream STAT3 signaling, and the maintenance of stemness, all of which contributed to acquired lapatinib resistance (Figures 1 & 3). Targeting IL-6 activity reduced the BTIC population and subsequent cell viability of these resistant cells. In contrast, stimulation of IL-6 rendered parental cells more resistant to lapatinib treatment (Figures 2 & 4). Thus, a switch of survival signaling from HER2 to IL-6 confers lapatinib resistance.

Mechanisms underlying resistance to lapatinib and trastuzumab are not always the same. Lapatinib resistance is mostly due to the switch of survival signaling from HER2 to other signals, such as AXL RTK [29], Src family kinase [30], etc. Moreover, the mechanism underlying lapatinib resistance may be different in HER2-positive breast cancer cells with or without estrogen receptor (ER) [31, 32]. Our study identified a new alternative survival signaling pathway, IL-6, for lapatinib resistance. However, we explored the mechanism of lapatinib resistance in HER2-positive breast cancer cells without ER expression. Whether this resistance mechanism is also observed in ER-positive breast cancer cells deserves further investigation. For trastuzumab resistance, the underlying mechanisms usually arise from the inability of trastuzumab to bind to HER2. Since trastuzumab is only able to bind to HER2 homodimers, HER2 forming heterodimers with other up-regulated RTKs, such as insulin-like growth factor 1 receptor [33, 34], MET [35] and HER3 [36] may confer trastuzumab resistance. This may explain why breast cancer patients are still sensitive to lapatinib treatment after acquiring trastuzumab resistance [13]. Therefore, lapatinib may sometimes be a successful treatment for trastuzumab resistance [37].

Interestingly, Korkaya et al., also demonstrated that constitutive activation of the IL-6 signaling loop contributes to trastuzumab resistance through enlargement of the BTIC population [38]. It seems that IL-6 activity may be a general cause of resistance to anti-HER2 therapy. Simultaneous targeting of IL-6 and HER2 may be a strategy not only to prevent, but also to overcome, the resistance to anti-HER2 therapy. Our previous study showed that long-term treatment of lapatinib in breast cancer cells with or without HER2 expression enhances NF-κB activation and subsequently results in the expression of NF-κB downstream genes, including *IL6* [26]. Furthermore, another more recent study by our group indicated that long-term treatment of lapatinib in triple-negative breast cancer cells increases *IL6* expression through miRNA-7-dependent activation of Raf-1 signaling [39]. How *IL6* expression is elevated in response to lapatinib resistance in this model awaits further clarification. On the other hand, whether other cytokine in addition to IL-6 is involved in lapatinib resistance deserves investigation. Building on these previous studies, this study not only explores a new mechanism underlying lapatinib resistance, but also provides a potential strategy to overcome this resistance via IL-6 inhibition.

## MATERIALS AND METHODS

### Cell lines and reagents

SkBr3 cells were maintained in Dulbecco's modified Eagle's medium/F12 medium supplemented with 10% fetal bovine serum (Logan, UT). Acquired lapatinib-resistant SkBr3/Lap#6 and SkBr3/Lap#9 cells were maintained in Dulbecco's modified Eagle's medium/F12 medium supplemented with 10% fetal bovine serum and 1 μM lapatinib. The QuickGene RNA cultured cell kit was from Kurabo (Osaka, JP). The RevertAid™ H Minus First Strand cDNA synthesis kit was purchased from Thermo Fisher Scientific (Waltham, MA). The ALDH activity colorimetric assay kit was from BioVision (Milpitas, CA). Si-control, si-*IL6* and si-*STAT3* as well as antibodies against Tubulin and Actin were purchased from Sigma-Aldrich (St. Louis, MO). TransIT-2020 transfection reagent from Mirus Bio LLC (Madison, WI) was used. Transwell chambers (24-well insert; pore size, 8mm) were purchased from Costar Corp. (Cambridge, MA). Human sIL-6 instant ELISA kit was from eBioscience (San Diego, CA). The ultra-low attachment multi-well plates were purchased from Corning (New York). The IL-6 recombinant protein was from Miltenyi Biotec (Bergisch Gladbach, Germany). The matrigel basement membrane matrix from BD Biosciences (East Rutherford, NJ) was used. The antibodies against phospho-Akt serine 473, Akt, phospho-STAT3 tyrosine 705 and phospho-ERK1/2 threonine 202/tyrosine 204 were from Cell Signaling (Danvers, MA). The antibody against STAT3 was purchased from Santa Cruz Biotechnology Inc. (Santa Cruz, CA). The antibody against ERK1/2 from Millipore (Darmstadt, Germany) was used. The IL-6 antibody was purchased from R&D Systems (Minneapolis, MN).

### Reverse transcription-polymerase chain reaction (RT-PCR)

The QuickGene RNA cultured cell kit was used for total RNA extraction according to manufacturer's instructions as described previously [40]. Briefly, 1 μg of RNA was used for reverse transcription using the RevertAid™ H Minus First Strand cDNA synthesis kit. PCR analysis of *IL6* mRNA expression was performed and visualized by 1% gel electrophoresis. *ACTIN* mRNA expression was used as an internal control. The primer sequences of IL-6 are as follows: forward 5′- ATGAACTCCTTCTCCACAAGCGC -3′; reverse 5′- GAAGAGCCCTCAGGATGGACTG -3′. The primer sequences of ACTIN are as follows: forward 5′- GGGTCAGAAGGATTCCTATG -3′; reverse 5′- GGTC TCAAACATGATCTGGG -3′.

### Enzyme linked immunosorbent assay (ELISA)

The production of IL-6 was detected by using human sIL-6 instant ELISA kit according to manufacturer's instructions. In brief, the number of microwell strips for the experiment was determined and the pellets were dissolved thoroughly by adding 100 μl of distilled water. Then, 50 μl samples, in duplicate, were added to designated wells and mixed with the contents. The microwell strips were covered by adhesive film and incubated at room temperature. After 3-hour incubation, microwell strips were mixed and washed thoroughly with 400 μl wash buffer (6 times). After the final wash, the excess buffer in the microwell strips was completely removed and 100 μl TMB substrate solution was added to each well, followed by incubation at room temperature for 1-10 minutes. The 100 μl stop solution was not added until the complete reaction was finished and the value of absorbance was determined immediately at 450 nm.

### Spheroid formation assay

Cells were trypsinized and washed with 1X PBS twice and with spheroid-inducing medium once. Then, cells at a density of 2 × 10^4^ cells/ml were seeded in an ultra-low attachment plate with spheroid-inducing medium. Cells were filtered and given new spheroid-inducing medium every week, allowing the formation of spheroids. The number of spheroids were then determined under microscope.

### Aldehyde dehydrogenase (ALDH) activity colorimetric assay

The ALDH activity was determined using ALDH activity colorimetric assay kit according to the manufacturer's instructions. Briefly, 2 × 10^6^ cells were rapidly homogenized with 200 μl of ice cold ALDH assay buffer for 10 minutes on ice. Then, cells were spun down at 12,000 rpm for 5 minutes to remove nuclei and insoluble material. 50 μl of collected supernatants was loaded into a 96-well plate and the final volume was adjusted to 50 μl with ALDH assay buffer. Then, 50 μl of reaction mix containing ALDH assay buffer, ALDH substrate mix, and acetaldehyde was added to each well. The mixture was incubated at room temperature for 5 minutes, followed by the measurement of absorbance at 450 nm.

### Transfection of siRNA

Cells at 60–80% of confluence were transfected with siRNA in a final concentration of 100 nM by using TransIT-2020 transfection reagent according to the manufacturer's instructions as described previously [41]. After 2-days, cells were subjected to further experiments, including the spheroid formation assay, the ALDH activity assay, *in vitro* transwell assay, and the cell viability assay. Cell lysates and ELISA assay for confirmation of gene silencing were harvested and performed 4 days after transfections.

### *In vitro* transwell migration and invasion assays

Cell migration and invasion were determined by transwell assay as described previously [40]. For the migration assay, cells at a density of 5 × 10^4^/well were seeded on the non-coated membrane of the upper chamber. For invasion assay, the membrane of the upper chamber was coated with matrigel for 1 hr before 5 × 10^4^ cells per well were seeded on the matrigel-coated membrane. After 48-hr incubation, cells were washed with 1X PBS once, followed by fixation with 4% formaldehyde. Thirty minutes later, cells were washed with 1X PBS once again and stained with 1% crystal violet in a solvent of 30% ethanol for 15-30 minutes at room temperature. Cells remaining on the upper chamber were removed using a cotton swab. The number of cells migrating or invading through the pores to the opposite side of the membrane was quantified.

### Cell viability assays

Cell viability assay was assessed using both the clonogenic and the 3-(4,5-dimethylthiazol-2-yl)-2,5-diphenyltetrazolium bromide (MTT) colorimetric assays. For the clonogenic assay, cells were re-seeded at a density of 2 × 10^4^/6-cm dish and allowed to grow for 2 weeks. Cell viability was detected and quantified by crystal violet staining. For MTT assay, 5 × 10^3^ cells/well were seeded on 96-well plates overnight and then pre-treated with indicated drugs. Three to four days later, relative cell amounts were determined by adding 1 μg/ml MTT to each well. Then, the medium was removed after 4-hour incubation and formazan was solubilized in 100 μl DMSO per well, followed by the measurement of absorbance at 570 nm.

### Statistical analysis

The statistical analysis was performed by Student's *t* test. *, *p<*0.05; **, *p<*0.01; ***, *p<*0.001 mean as compared to each control groups.
